# A Software Tool for Estimating Uncertainty of Bayesian Posterior Probability for Disease

**DOI:** 10.3390/diagnostics14040402

**Published:** 2024-02-12

**Authors:** Theodora Chatzimichail, Aristides T. Hatjimihail

**Affiliations:** Hellenic Complex Systems Laboratory, Kostis Palamas 21, 66131 Drama, Greece; tc@hcsl.com

**Keywords:** Bayesian diagnosis, Bayesian inference, prior probability, posterior probability, likelihood, parametric distribution, probability density function, uncertainty, combined uncertainty, measurement uncertainty, sampling uncertainty, confidence intervals, diabetes mellitus, fasting plasma glucose, oral glucose tolerance test

## Abstract

The role of medical diagnosis is essential in patient care and healthcare. Established diagnostic practices typically rely on predetermined clinical criteria and numerical thresholds. In contrast, Bayesian inference provides an advanced framework that supports diagnosis via in-depth probabilistic analysis. This study’s aim is to introduce a software tool dedicated to the quantification of uncertainty in Bayesian diagnosis, a field that has seen minimal exploration to date. The presented tool, a freely available specialized software program, utilizes uncertainty propagation techniques to estimate the sampling, measurement, and combined uncertainty of the posterior probability for disease. It features two primary modules and fifteen submodules, all designed to facilitate the estimation and graphical representation of the standard uncertainty of the posterior probability estimates for diseased and non-diseased population samples, incorporating parameters such as the mean and standard deviation of the test measurand, the size of the samples, and the standard measurement uncertainty inherent in screening and diagnostic tests. Our study showcases the practical application of the program by examining the fasting plasma glucose data sourced from the National Health and Nutrition Examination Survey. Parametric distribution models are explored to assess the uncertainty of Bayesian posterior probability for diabetes mellitus, using the oral glucose tolerance test as the reference diagnostic method.

## 1. Introduction

### 1.1. Diagnosis in Medicine

Diagnosis in medicine fundamentally involves identifying the unique characteristics of a disease and distinguishing it from other conditions with similar presentations. The term “diagnosis”, originating from the Greek word “διάγνωσις” meaning “discernment” [1], emphasizes the critical role of distinguishing between healthy and diseased states in individuals. Diagnostic tests are essential in classifying individuals based on their health status. However, the reliance on a singular threshold for diagnosis across a range of data points introduces uncertainty, owing to the overlapping probability distributions of a measurand in both healthy and diseased populations [2]. While traditional diagnostic methods have been broadly effective, they may not fully encompass the diversity of disease manifestations, particularly across varied groups of people [3].

As underlined previously [2], Bayesian inference represents a paradigm shift in the field of medical diagnosis, offering a robust framework for integrating various sources of information to make probabilistic assessments. At its core, Bayesian inference relies on the Bayes’ theorem for updating beliefs in light of new evidence, integrating prior disease probabilities with the distribution of diagnostic measurands to calculate posterior probabilities for disease [4,5,6,7]. This approach enables a more comprehensive probabilistic assessment, evaluation of the information conveyed by diagnostic measurements, and a personalized patient approach [3,8].

Historically, the application of Bayesian methods in medicine has undergone significant evolution. Despite facing several challenges and being met with skepticism, these methods have gradually gained acceptance.

#### 1.1.1. Bayes’ Theorem in Medical Diagnostics

Bayes’ theorem, a fundamental principle in probability theory [5], forms a connection between the direct probability P(H|E) of a hypothesis H given specific data E, and the inverse probability P(E|H) of data E given the hypothesis H [9]. In medical diagnostics, Bayes’ theorem is instrumental in transforming the prior probability for disease into a posterior probability following diagnostic tests [4].

#### 1.1.2. Challenges in Applying Bayesian Inference

The application of Bayesian inference in diagnostics, however, faces significant challenges.

##### Computational Complexity

The computational complexity of Bayesian inference requires considerable resources.

##### Statistical Distributions in Diagnostics

A major challenge involves comprehensively understanding the statistical distributions of diagnostic test measurands in both diseased and nondiseased populations [10]. Calculation of posterior probabilities requires probability density functions (PDF) for the measurands in these populations. The normal distribution, often used for its simplicity, may not be suitable for measurands with non-normal characteristics like skewness or multimodality. Critical evaluation and potential adoption of alternative distributions are necessary for more accurate Bayesian diagnostic methods [10,11,12]. Bayesian Diagnosis, our previously published software, addresses this challenge [2].

##### Uncertainty of Bayesian Posterior Probabilities

Another significant challenge involves estimating the uncertainty associated with Bayesian posterior probabilities in disease diagnosis. This uncertainty can substantially affect their clinical utility. Despite its critical importance, the task of estimating, evaluating, and mitigating uncertainty in Bayesian diagnostic test interpretation has seldom been addressed in medical literature [13]. To confront this issue, we have developed Bayesian Diagnostic Uncertainty, a software tool for the estimation of uncertainty in Bayesian diagnosis, which is presented in detail in this study.

Both Bayesian Diagnostic Uncertainty and Bayesian Diagnosis, enhance the applicability of Bayesian methods in medical diagnostics.

#### 1.1.3. Quantifying Uncertainty in Diagnostics

Uncertainty can be quantified and is often expressed probabilistically [14].

##### Combined Uncertainty

In the context of Bayesian posterior probability for disease, we consider two main components of combined uncertainty:

##### Measurement Uncertainty

This reflects the inherent variability in measurement processes and is defined as a parameter characterizing the dispersion of values that could reasonably be attributed to the measurand [15]. While crucial for laboratory quality assurance, the impact of measurement uncertainty on clinical decision-making and outcomes is often underexplored and rarely quantified [16,17]. Emerging research focuses on its effects on misclassification [18] and on diagnostic accuracy measures [19].

##### Sampling Uncertainty

The variability in sampling contributes to the uncertainty of posterior probability for disease [20], and it is essential in evaluating diagnostic methods.

## 2. Methods

### 2.1. Computational Methods

#### 2.1.1. Bayes’ Theorem

Bayes’ theorem calculates the posterior probability $P\left(D|T\right)$ of a disease D given a test result T=x and a parameter vector ***θ***, as follows:

$$P\left(D|T\right)=\frac{f_{D}\left(x|\theta \right)r}{f_{D}\left(x|\theta \right)r+f_{\bar{D}}\left(x|\theta \right)\left(1-r\right)}$$

Here r denotes the prior probability for disease, $f_{D}\left(x|\theta \right)$ the PDF in disease presence, while $f_{\bar{D}}\left(x;\;\theta \right)$ denotes the PDF in its absence (refer to Appendix A.1 for details).

#### 2.1.2. Parametric Distributions

Parametric statistics operate under the assumption that data from a population can be accurately represented by a probability distribution with a fixed set of parameters [21]. The program supports the following parametric distributions:

1. Normal distribution
2. Lognormal distribution
3. Gamma distribution.

#### 2.1.3. Uncertainty Quantification

Uncertainty of input parameters can manifest as standard uncertainty $u\left(x\right)$ , the standard deviation of x , and expanded uncertainty $U\left(x\right)$ , a range around x encompassing x with a probability p [16].

##### Measurement Uncertainty

Measurement uncertainty is computed following guidelines in the “Guide to the expression of uncertainty in measurement” (GUM) [15] and “Expression of measurement uncertainty in laboratory medicine” [16]. Bias is considered a component of this uncertainty [22].

The relationship between the standard measurement uncertainty $u\left(x\right)$ to the value of the measurand x , is generally expressed as:

$$u_{m}\left(x\right)=\sqrt{b_{0}^{2}+b_{1}^{2}x^{2}}$$

where $b_{0}$ is a constant and $b_{1}$ is a proportionality constant.

If needed, it is approximated linearly as:

$$u_{m}\left(x\right)≅b_{0}+b_{1}x$$

The general approach to estimating the coefficients of the above equations is delineated in Appendix A5 of “Quantifying Uncertainty in Analytical Measurement” [23].

##### Sampling Uncertainties of Means and Standard Deviations

If $m_{P}$ and $s_{P}$ are the mean and standard deviation of a measurand in a population sample of size $n_{P}$ , then the standard sampling uncertainties of $m_{P}$ and $s_{P}$ are estimated as:

$$u_{s}\left(m_{P}\right)≅\frac{s_{P}}{\sqrt{n_{P}}}$$

$$u_{s}\left(s_{P}\right)≅\frac{s_{p}}{\sqrt{2\left(n_{P}-1\right)}}$$

using the central limit theorem and the chi-square distribution [24,25,26].

##### Sampling Uncertainty of Prior Probability for Disease 

If $n_{D}$ and $n_{\bar{D}}$ are the respective numbers of diseased and nondiseased in a population sample, then the standard uncertainty of the prior probability for disease $r=\frac{n_{D}}{n_{\bar{D}}+n_{D}}$ is estimated as:

$$u_{s}\left(r\right)≅\sqrt{\frac{\left(2+n_{\bar{D}})(2+n_{D}\right)}{{\left(4+n_{\bar{D}}+n_{D}\right)}^{3}}}$$

using the Agresti–Coull adjustment of the Waldo interval [27].

##### Combined Uncertainty of Posterior Probability for Disease 

The standard combined uncertainty $u_{c}\left(x\right)$ of posterior probability for disease is computed via uncertainty propagation rules, employing a first-order Taylor series approximation [28] (refer to Supplementary File S2). 

When there are *l* components of uncertainty, with standard uncertainties $u_{i}\left(x\right)$ , then:

$$u_{c}\left(x\right)=\sqrt{\overset{l}{\underset{i=1}{\sum }}u_{i}{\left(x\right)}^{2}}$$

#### 2.1.4. Expanded Uncertainty of Posterior Probability for Disease 

When there are *l* components of uncertainty, with standard uncertainties $u_{i}\left(x\right)$ and $v_{i}$ degrees of freedom, then the effective degrees of freedom $v_{eff}$ of the combined uncertainty $u_{c}\left(x\right)$ are obtained from the Welch–Satterthwaite formula [29,30]:

$$v_{eff}\left(x\right)≅\frac{u_{c}{\left(x\right)}^{4}}{\sum_{i=1}^{l}\frac{u_{i}{\left(x\right)}^{4}}{v_{i}}}$$

If $v_{min}$ the minimum of $v_{1},v_{2},\ldots ,v_{l}$ , then:

$$v_{min}\leq v_{eff}\left(x\right)\leq \overset{l}{\underset{i=1}{\sum }}v_{i}$$

If $F_{v}\left(z\right)$ is the Student’s *t*-distribution cumulative distribution function with v degrees of freedom and $u_{c}\left(x\right)$ is the standard combined uncertainty of posterior probability for disease, its expanded combined uncertainty $U_{c}\left(x\right)$ at a confidence level p is:

$$U_{c}\left(x\right)≅\left(F_{v}^{-1}\left(\frac{1-p}{2}\right)u_{c}\left(x\right),F_{v}^{-1}\left(\frac{1+p}{2}\right)u_{c}\left(x\right)\right)$$

The confidence interval of x at the same confidence level p is approximated as:

$$CI_{p}\left(x\right)≅\left(x+F_{v}^{-1}\left(\frac{1-p}{2}\right)u_{c}\left(x\right),x+F_{v}^{-1}\left(\frac{1+p}{2}\right)u_{c}\left(x\right)\right)$$

The confidence intervals of the posterior probability for disease were truncated to the [0, 1] range.

### 2.2. The Software

#### 2.2.1. Program Overview

**Figure 1:**
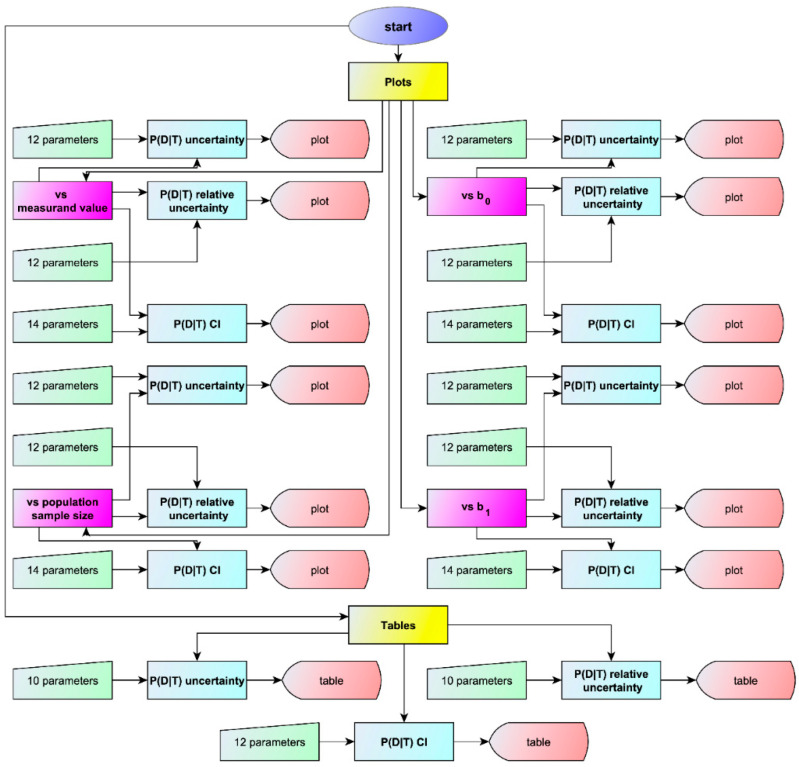
A simplified flowchart of the program Bayesian Diagnostic Uncertainty with the number of input parameters and of output types for each submodule.

To facilitate the estimation of the uncertainty of Bayesian posterior probability for disease, the software program Bayesian Diagnostic Uncertainty was developed in Wolfram Language, using Wolfram Mathematica^®^ Ver. 13.3 (Wolfram Research, Inc., Champaign, IL, USA). Bayesian Diagnostic Uncertainty was designed to estimate and plot the standard sampling, measurement, and combined uncertainty and the confidence intervals of the Bayesian posterior probability for disease of a screening or diagnostic test (See Figure 1).

This interactive program is freely available as a Wolfram Language notebook (.nb) (Supplementary File S2: BayesianUncertainty.nb). It can be run on Wolfram Player^®^ (Wolfram Research, Inc., Champaign, IL, USA (2023)) or Wolfram Mathematica^®^ (see Appendix A.2). Due to the complexity of the calculations required, it is computationally intensive.

#### 2.2.2. Input Parameters

The program allows for the definition of three parametric distributions of a measurand for the diseased and nondiseased populations.

Distribution Selection: The user selects the type of distribution of each population from a predefined list:

1. Normal distribution
2. Lognormal distribution
3. Gamma distribution.

Definition of Statistical Parameters: For each population, the user defines its size *n*, the mean *μ*, and the standard deviation *σ* of the measurand.

##### Measurement Uncertainty

The user selects a linear or nonlinear equation of the measurement uncertainty versus the value *x* of the measurand and defines the constant contribution $b_{0}$ to the standard measurement uncertainty, the proportionality constant $b_{1}$ , and the number of quality control samples that have been analyzed for its estimation.

#### 2.2.3. Output Specifications

##### Visualizations

The program generates a series of plots designed to elucidate various uncertainty measures and statistics:

1. Uncertainty of posterior probability for disease: Plots are generated to show the standard sampling, measurement, and combined uncertainty of the posterior probability for disease.
2. Relative uncertainty of posterior probability for disease: Plots are generated to show the relative standard sampling, measurement, and combined uncertainty of the posterior probability for disease.
3. Confidence intervals of posterior probability for disease: Plots are generated to show the confidence intervals of the posterior probability for disease, for a user defined confidence level.

##### Tables

For each combination of parametric distributions of the diseased and nondiseased populations, the program tabulates for a user defined measurand value:

1. The standard sampling, measurement, and combined uncertainty of the posterior probability for disease.
2. The relative standard sampling, measurement, and combined uncertainty of the posterior probability for disease.
3. The confidence intervals of the posterior probability for disease for a user defined confidence level.

**Figure 2:**
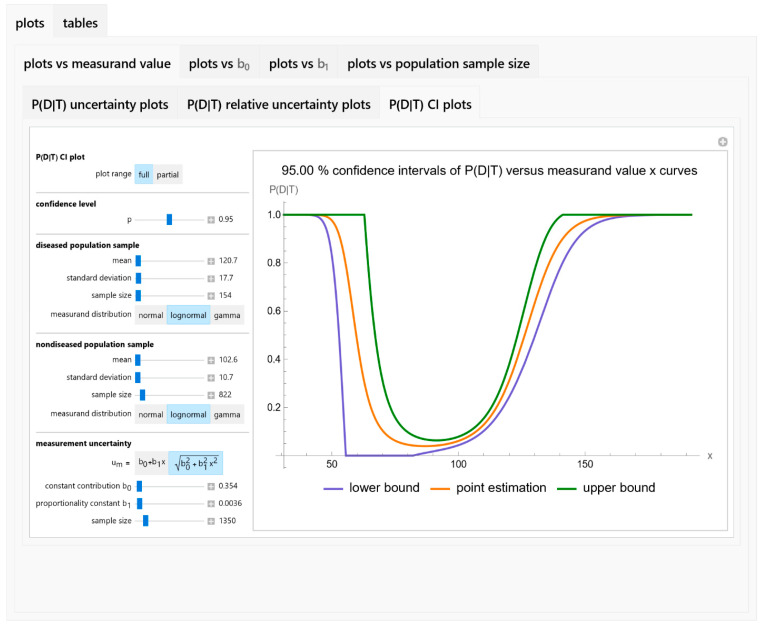
A screenshot of the program Bayesian Diagnostic Uncertainty.

By providing this comprehensive set of input parameters and output specifications (see Figure 2), the program offers a robust platform for exploring the uncertainty in Bayesian diagnosis of disease using parametric distributions of medical diagnostic measurands.

## 3. Illustrative Case Study

To demonstrate the application of the program, fasting plasma glucose (FPG) was used as the diagnostic test measurand for the Bayesian diagnosis of diabetes mellitus (From now on, when mentioning “diabetes”, we are referring to diabetes mellitus). The oral glucose tolerance test (OGTT) was used as the reference diagnostic method. A diagnosis of diabetes was confirmed if the plasma glucose value was equal to or greater than 200 mg/dL, measured two hours after oral administration of 75 g of glucose [31], during an OGTT (2-h PG). The study population was confined to individuals aged between 70 and 80 years, a decision guided by the well-documented strong correlation between age and the prevalence of diabetes [32].

National Health and Nutrition Examination Survey (NHANES) data from participants was retrieved for the period from 2005 to 2016 (*n* = 60,936) [33]. NHANES is a series of studies designed to evaluate the health and nutritional status of adults and children in the United States. 

The inclusion criteria for participants were:

1. Valid FPG and OGTT results (*n* = 13,836).
2. A negative response to NHANES question DIQ010 regarding a diabetes diagnosis [34] (*n* = 13,465).
3. Age 70–80 years (*n* = 976).

Participants with a 2-h PG measurement ≥200 mg/dL were considered diabetic (*n* = 154).

The prior probability for diabetes was estimated as:

$$v≅\frac{154}{976}=0.158$$

The statistics of the FPG datasets are presented in Table 1 (Hereafter, FPG and its uncertainty are expressed in mg/dL).

Lognormal distributions were estimated to model FPG measurements in diabetic and nondiabetic participants, using the maximum likelihood estimation method [35]. The respective distributions, parametrized for their means $\mu_{D}$ and $\mu_{\bar{D}}$ , and standard deviations $\sigma_{D}$ and $\sigma_{\bar{D}}$ , were the following:

$$L_{D}=Lognormal\left(\mu_{D},\sigma_{D}\right)=Lognormal\left(120.671,17.720\right)\;L_{\bar{D}}=Lognormal\left(\mu_{\bar{D}},\sigma_{\bar{D}}\right)=Lognormal\left(102.642,10.653\right)$$

The NHANES quality control data of the FPG measurements was retrieved for the same period (2005–2016). In total, 1350 QC samples had been analyzed. The weighted nonlinear least squares analysis [36] yielded the following function relating the standard measurement uncertainty $u_{m}\left(x\right)$ to the measurement value, x :

$$u_{m}\left(x\right)=\sqrt{b_{0}^{2}+b_{1}^{2}x^{2}}=\sqrt{0.7501+0.00012x^{2}}$$

where $b_{0}=0.866$ and $b_{1}=0.0109$ .

The means of the standard measurement uncertainty of FPG of the included diabetic and nondiabetic participants were estimated as:

$${\hat{u}}_{D}≅1.586\;\mathrm{mg}/\mathrm{dL}\;{\hat{u}}_{\bar{D}}≅1.028\;\mathrm{mg}/\mathrm{dL}$$

Consequently, the distributions of the measurands, assuming negligible uncertainty, were estimated as:

$$l_{D}≅Lognormal\left(\mu_{D},\sqrt{\sigma_{D}^{2}-{\hat{u}}_{D}^{2}}\right)≅Lognormal\left(120.671,17.720\right)\;$$

$$l_{\bar{D}}≅Lognormal\left(\mu_{\bar{D}},\sqrt{\sigma_{\bar{D}}^{2}-{\hat{u}}_{\bar{D}}^{2}}\right)≅Lognormal\left(102.642,10.653\right)$$

Table 2 displays the descriptive statistics of the estimated lognormal distributions of the diabetic and nondiabetic populations, including the respective *p*-values of the Cramér–von Mises goodness-of-fit test [37].

**Figure 3:**
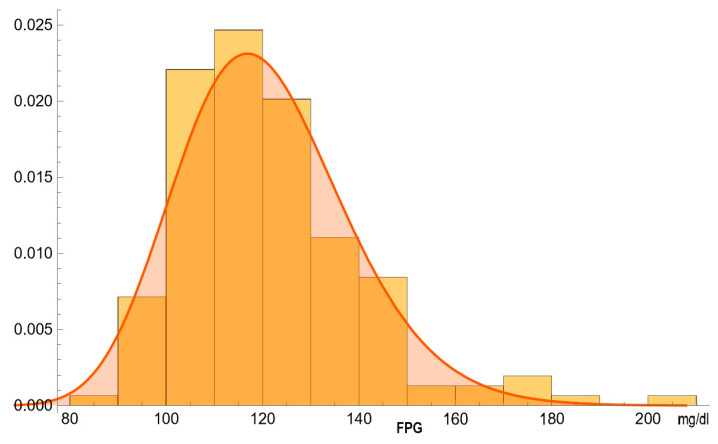
The estimated PDF of the FPG (mg/dL) in diabetic participants, assuming a lognormal distribution and negligible measurement uncertainty, and the histogram of the respective NHANES dataset, with the parameters of the distribution in Table 2.

**Figure 4:**
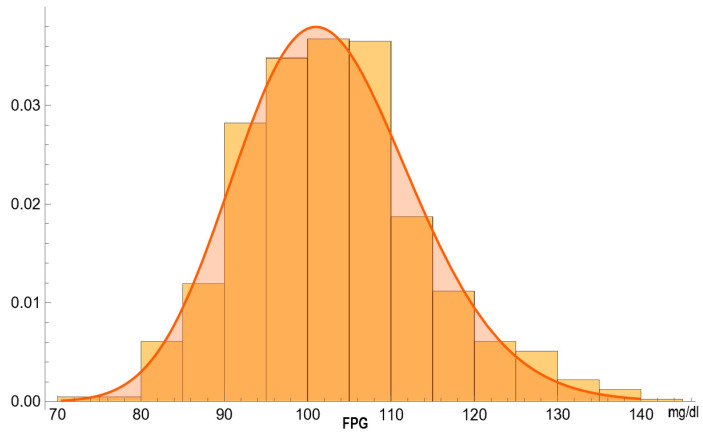
The estimated PDF of the FPG (mg/dL) in nondiabetic participants, assuming a lognormal distribution and negligible measurement uncertainty, and the histogram of the respective NHANES dataset, with the parameters of the distribution in Table 2.

Figure 3 and Figure 4 show the estimated PDFs of FPG in the diabetic and nondiabetic populations, assuming a lognormal distribution and negligible measurement uncertainty, and the histograms of the respective NHANES datasets.

Likelihoods and posterior probabilities were estimated accordingly.

## 4. Results

Using the settings of Table 3, the program generated the plots of Figure 5, Figure 6, Figure 7, Figure 8, Figure 9, Figure 10, Figure 11, Figure 12, Figure 13, Figure 14, Figure 15, Figure 16 and Figure 17 and the tables of Figure 17, Figure 18 and Figure 19. 

**Figure 5:**
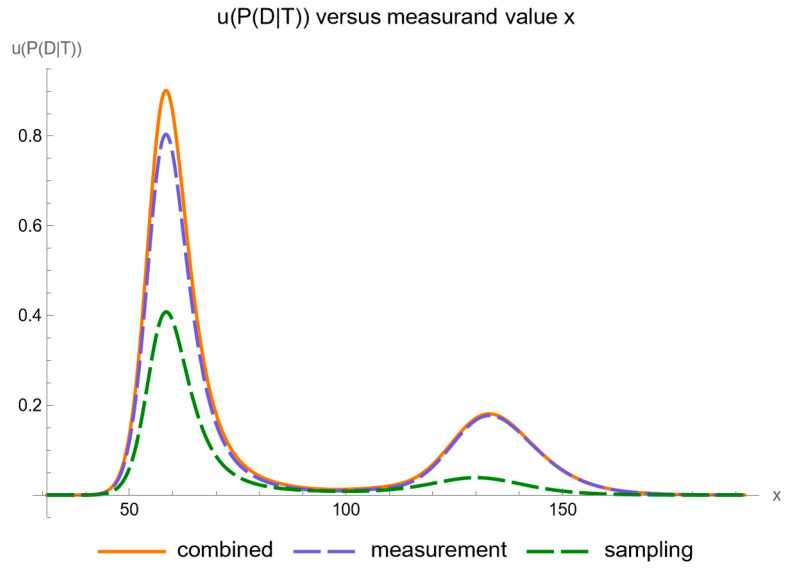
Standard sampling, measurement, and combined uncertainty of the posterior probability for diabetes versus FPG curve plot, with the settings of the program in Table 2.

**Figure 6:**
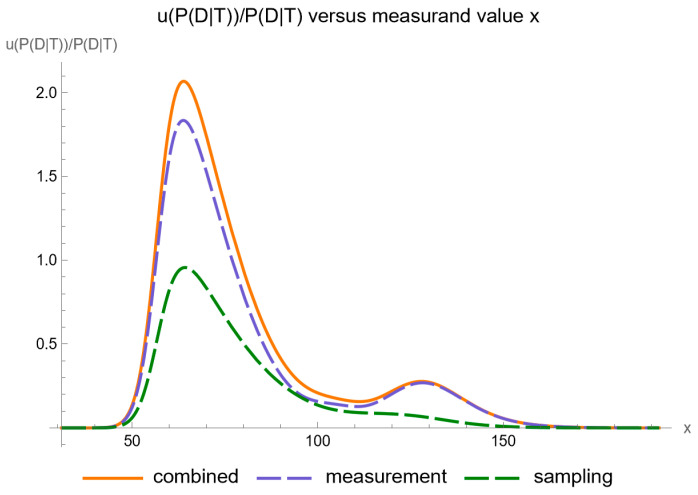
Relative standard sampling, measurement, and combined uncertainty of the posterior probability for diabetes versus FPG curve plot, with the settings of the program in Table 2.

Figure 5 shows the plots of the standard sampling, measurement, and combined uncertainty of posterior probability for diabetes versus FPG, while Figure 6 shows the respective plots of the relative standard uncertainty.

**Figure 7:**
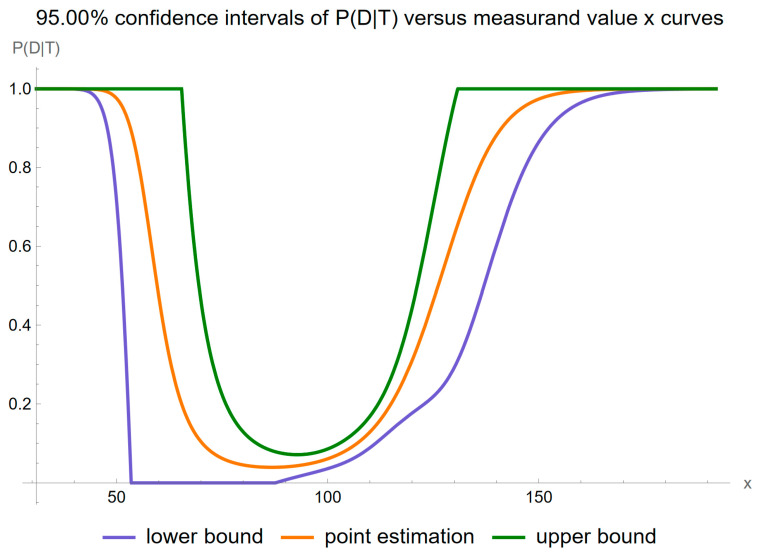
Confidence intervals of the posterior probability for diabetes versus FPG curves plot, with the settings of the program in Table 2.

Figure 7 shows the plots of the confidence intervals of posterior probability for diabetes versus FPG for a confidence level p=0.95 .

Assessing the combined standard uncertainty of the posterior probability for diabetes, we note the following:

1. It is substantially affected by measurement uncertainty of FPG.
2. Two local maxima are observed, corresponding to the regions near the steepest segments of the posterior probability curve, which exhibits an approximately double sigmoidal configuration. These maxima are quantitatively defined as following:
  2.1. At an FPG value of 58.7 mg/dL, the posterior probability for disease is equal to 0.585, while the combined standard uncertainty is equal to 0.893.
  2.2. At an FPG value of 133.2 mg/dL, the posterior probability for disease is equal to 0.725, while the combined standard uncertainty is equal to 0.182.

This pattern of local maxima is indicative of heightened uncertainty in the regions where the posterior probability curve demonstrates its most pronounced inflections. The confidence intervals are affected accordingly.

Assessing the relative combined standard uncertainty of the posterior probability for diabetes, we note that two local maxima are observed as well, quantitatively defined as following:

1. At an FPG value of 64.1 mg/dL, the posterior probability for disease is equal to 0.257, while the relative combined standard uncertainty is equal to 2.044.
2. At an FPG value of 128.1 mg/dL, the posterior probability for disease is equal to 0.561, while the relative combined standard uncertainty is equal to 0.278.

**Figure 8:**
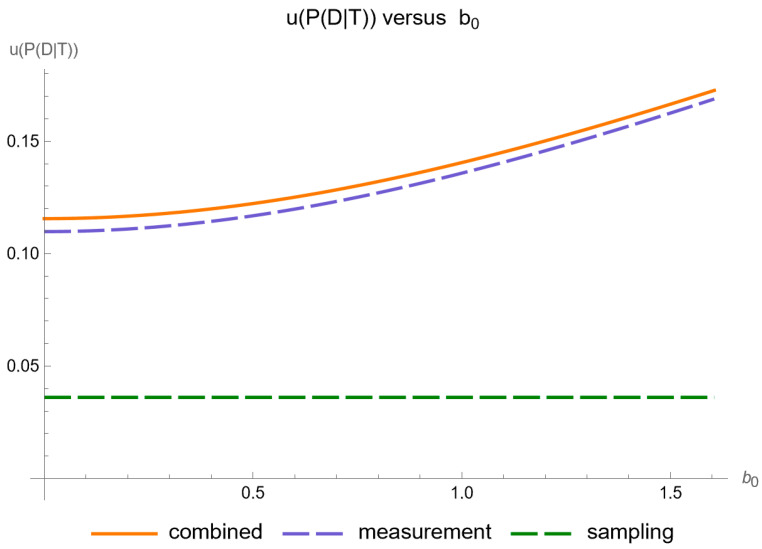
Standard sampling, measurement, and combined uncertainty of the posterior probability for diabetes versus measurement uncertainty constant contribution $b_{0}$ curve plot, with the settings of the program in Table 2.

**Figure 9:**
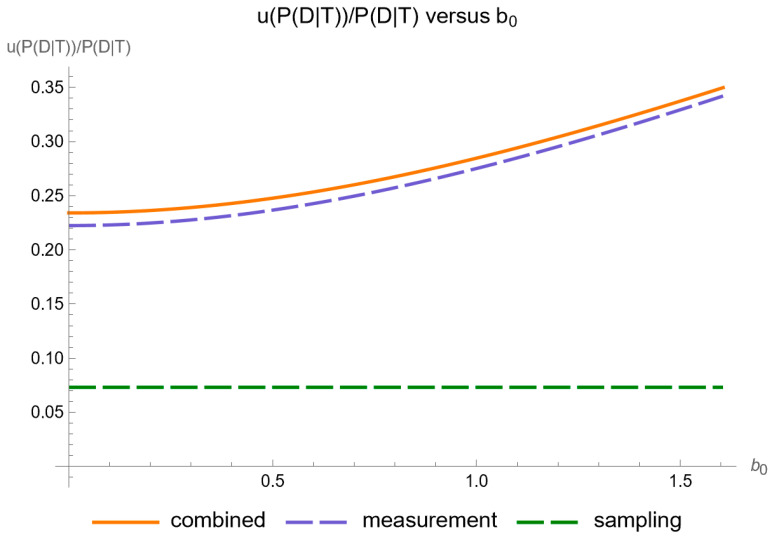
Relative standard sampling, measurement, and combined uncertainty of the posterior probability for diabetes versus measurement uncertainty constant contribution $b_{0}$ curve plot, with the settings of the program in Table 2.

Figure 8 shows the plots of the standard sampling, measurement, and combined uncertainty of posterior probability for diabetes versus the constant contribution $b_{0}$ of measurement uncertainty of FPG, while Figure 9 shows the respective plots of the relative standard uncertainty.

**Figure 10:**
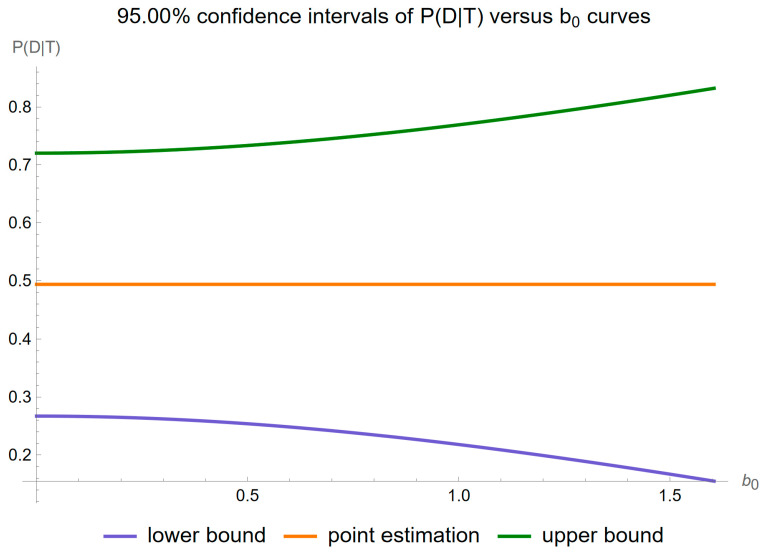
Confidence intervals of the posterior probability for diabetes versus measurement uncertainty constant contribution $b_{0}$ curves plot, with the settings of the program in Table 2.

Figure 10 shows the plots of the confidence intervals of posterior probability for diabetes versus the constant contribution $b_{0}$ of measurement uncertainty of FPG, for a confidence level p=0.95 .

**Figure 11:**
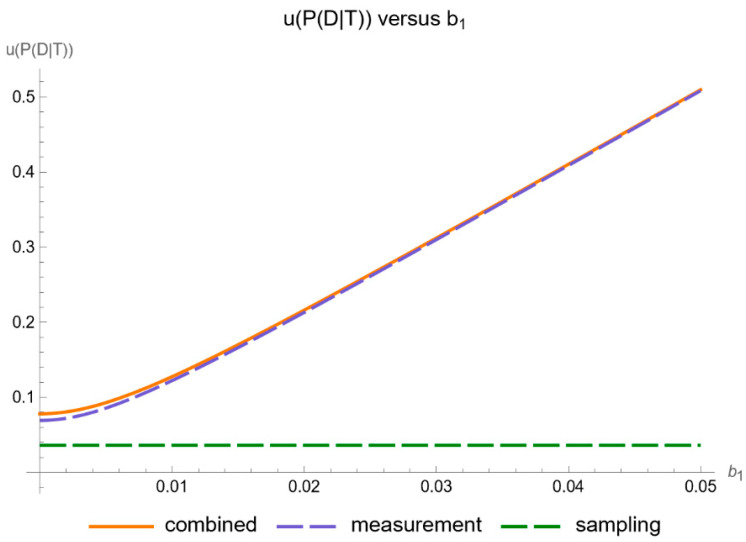
Standard sampling, measurement, and combined uncertainty of the posterior probability for diabetes versus measurement uncertainty proportionality constant $b_{1}$ curve plot, with the settings of the program in Table 2.

**Figure 12:**
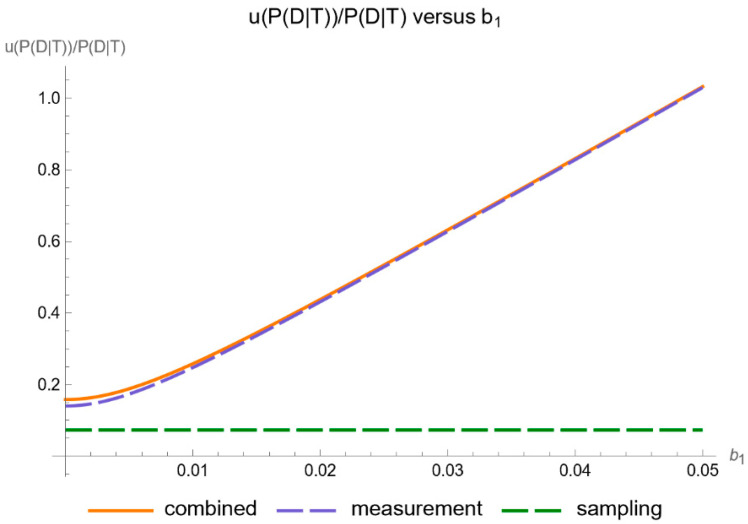
Relative standard sampling, measurement, and combined uncertainty of the posterior probability for diabetes versus measurement uncertainty proportionality constant $b_{1}$ curve plot, with the settings of the program in Table 2.

Figure 11 shows the plots of the standard sampling, measurement, and combined uncertainty of posterior probability for diabetes versus the proportionality constant $b_{1}$ of measurement uncertainty of FPG, while Figure 12 shows the respective plots of the relative standard uncertainty.

**Figure 13:**
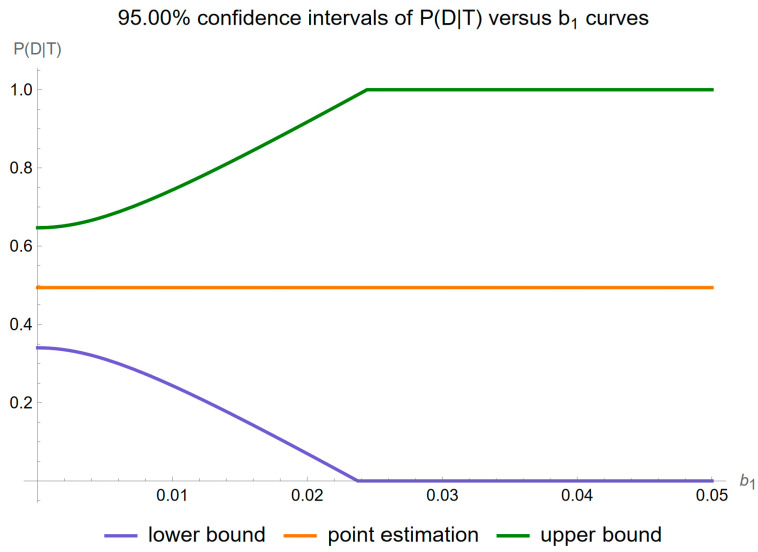
Confidence intervals of the posterior probability for diabetes versus measurement uncertainty proportionality constant $b_{1}$ curves plot, with the settings of the program in Table 2.

Figure 13 shows the plots of the confidence intervals of posterior probability for diabetes versus the proportionality constant $b_{1}$ of measurement uncertainty of FPG for a confidence level p=0.95 .

**Figure 14:**
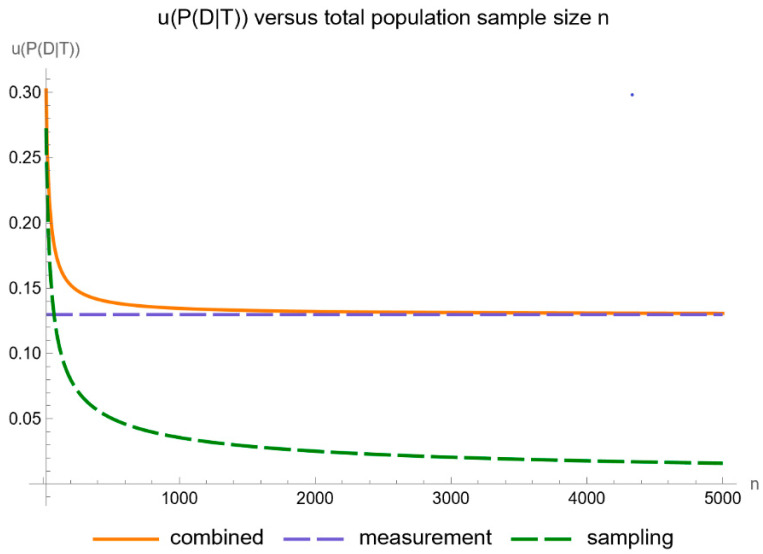
Standard sampling, measurement, and combined uncertainty of the posterior probability for diabetes versus total population sample size *n* curve plot, with the settings of the program in Table 2.

**Figure 15:**
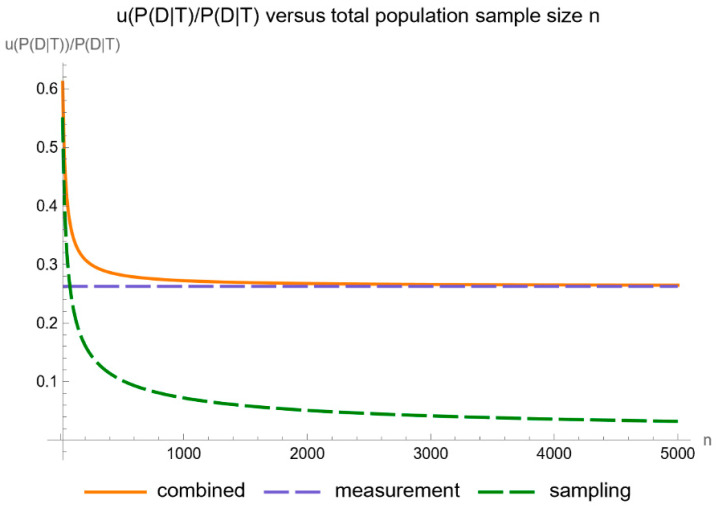
Relative standard sampling, measurement, and combined uncertainty of the posterior probability for diabetes versus total population sample size *n* curve plot, with the settings of the program in Table 2.

Figure 14 shows the plots of the standard sampling, measurement, and combined uncertainty of posterior probability for diabetes versus the total population size *n*, while Figure 15 shows the respective plots of the relative standard uncertainty.

**Figure 16:**
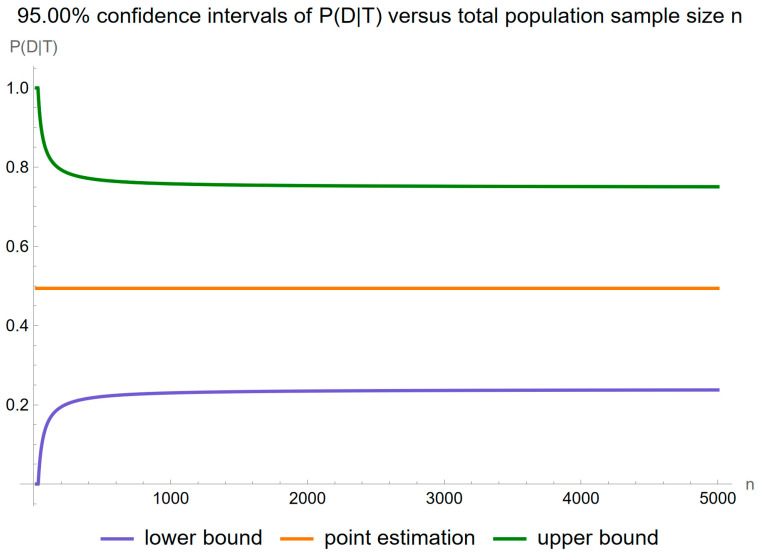
Confidence intervals of the posterior probability for diabetes versus total population sample size *n* curves plot, with the settings of the program in Table 2.

Figure 16 shows the plots of the confidence intervals of posterior probability for diabetes versus the total population size *n*, for a confidence level p=0.95 .

As anticipated, the impact of sampling uncertainty decreases markedly as the size of the population sample increases.

**Figure 17:**
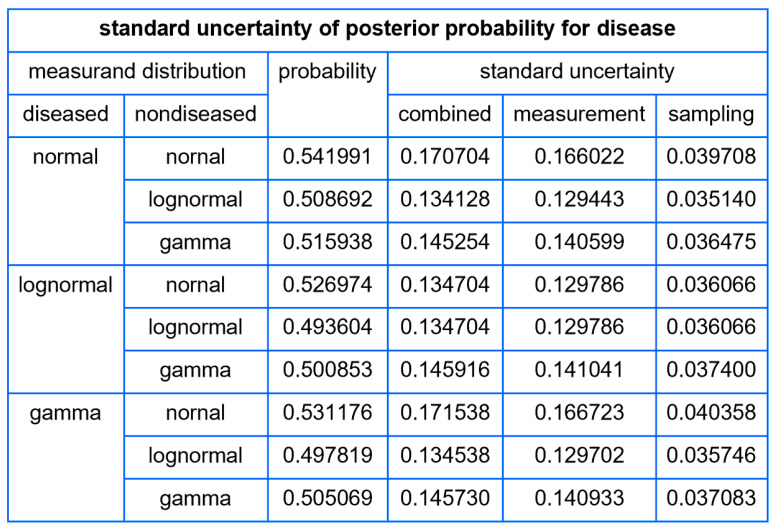
Table of the standard sampling, measurement, and combined uncertainty of the posterior probability for diabetes, with the settings of the program in Table 2.

**Figure 18:**
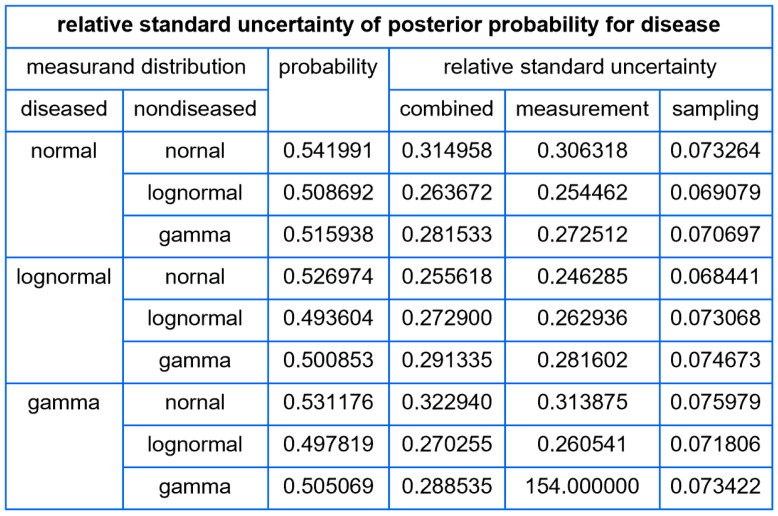
Table of the relative standard sampling, measurement, and combined uncertainty of the posterior probability for diabetes, with the settings of the program in Table 2.

Figure 17 shows a table of the standard sampling, measurement, and combined standard uncertainty of posterior probability for diabetes for FPG value equal to 126 mg/dL, while Figure 18 shows a table of the respective values of relative standard uncertainty.

Figure 18 shows the confidence intervals of posterior probability for diabetes for FPG value equal to 126 mg/dL and confidence level p=0.95 .

The tables distinctly demonstrate the considerable magnitude of uncertainty and relative uncertainty associated with the posterior probability for diabetes at an FPG level of 126 mg/dL, the established threshold for the diagnosis of diabetes. Furthermore, the posterior probabilities delineated in the tables suggest a limited concordance between the classification criteria of diabetes derived from the OGTT and FPG tests [31], as found previously in existing literature [38].

## 5. Discussion

### 5.1. Reevaluation of Traditional Diagnostic Methods

Traditional diagnostic methods rely on the use of predetermined thresholds; however, this often fails to consider the complexities of disease pathology. While this has been historically effective, it may lack the ability to offer a holistic approach in today’s patient-centered medicine, where personalized care is paramount [39]. The evolving nature of diseases and shifts in patient demographics increase the complexity of the diagnostic process, pushing the boundaries of conventional methodologies. In this challenging context, Bayesian inference emerges as an alternative approach, offering probabilistic evaluations that can adapt to the individual patient profiles [2,3].

Nevertheless, estimating the uncertainty of posterior probabilities within Bayesian inference remains a pivotal challenge [13]. This issue is critically important in the context of diagnostic and screening tests for life-threatening conditions or those associated with considerable morbidity risk. It underscores the need for well-informed clinical judgments and comprehensive uncertainty evaluation in medical decision-making. Key examples include:

1. Cardiac troponin for diagnosing myocardial injury and infarction [40];
2. Natriuretic peptides for the diagnosis of heart failure [41];
3. D-dimer for diagnosing thromboembolic events [42];
4. FPG, OGTT, and glycated hemoglobin (HbA1c) for diagnosing diabetes [31];
5. OGTT for the diagnosis of gestational diabetes [43];
6. Thyroid stimulating hormone (TSH), free serum triiodothyronine (T_3_), and free serum thyroxine (T_4_) for diagnosing thyroid dysfunction [44];
7. Protein-to-creatinine ratio for the diagnosis of preeclampsia [45];
8. Creatinine or cystatin C derived glomerular filtration rate (GFR), and albuminuria for diagnosing chronic kidney disease [46].

The ability to quantify this uncertainty is not a purely academic concern but also a practical necessity in improving diagnosis and patient outcomes.

To address this, our software explores the sampling, measurement, and combined uncertainty of Bayesian posterior probabilities. This exploration is not only vital for enhancing clinical decision-making but also plays a significant role in the fields of quality and risk management in laboratory medicine [47]. Additionally, it may contribute to the design and implementation of test accuracy studies [48,49]. As mentioned in Section 1, despite the extensive body of research on Bayesian diagnosis and uncertainty as separate entities, the intersection of these two areas remains relatively unexplored [50,51].

The illustrative case study, focusing on individuals aged 70 to 80 years, was designed to mitigate age-related variations in disease prevalence. This focus exemplifies the considerations required in modern diagnostics, where factors such as age, genetics, and lifestyle choices should be accounted for in the diagnostic equation.

Our software manages through its analysis of sampling, measurement, and combined uncertainty (as illustrated in Figure 5, Figure 8, Figure 11, Figure 14 and Figure 17), relative uncertainty (Figure 6, Figure 9, Figure 12, Figure 15 and Figure 18) and the corresponding confidence limits (Figure 7, Figure 10, Figure 13, Figure 16 and Figure 19), to display its versatility in addressing these diagnostic challenges. Although the software’s calculations are highly sophisticated, its user-friendly interface renders it an effective tool for medical researchers and professionals.

The case study from Section 4 highlights the substantial impact of combined uncertainty on the diagnostic process. This finding emphasizes the predominant role of measurement uncertainty, and thus stresses the demanding path toward enhancing diagnostic accuracy. By improving the analytical methods of screening and diagnostic tests, the medical community could achieve more accurate diagnosis, leading to more effective and tailored patient care.

Looking ahead, future research should focus on improving the estimations of the uncertainty of posterior probabilities under a diverse array of clinically relevant parameter settings. To transition from research into practical application, it is necessary to focus on clinical decision analysis, studies on cost-effectiveness, and research on quality of care, which includes conducting implementation studies [48]. Such efforts are necessary in addressing the complex issues in diagnostic medicine and finding new and effective approaches to tackle ongoing challenges.

### 5.2. Limitations of the Program

This program’s limitations, which provide paths for further research, include:

1. Underlying assumptions:
  1.1. The existence of “gold standards” in diagnostics. If a “gold standard” does not exist, there are alternative approaches for classification [52,53,54].
  1.2. The hypothesis of parametric distribution of measurements or their transformations. However, existing literature underlines the robustness of nonparametric techniques in capturing complex data distributions [55].
  1.3. The generally accepted bimodality of the measurands, although unimodal distributions could be considered [56,57].

If these assumptions are not valid, the program may underestimate the standard uncertainty of the posterior probability for disease.

2. The use of first-order Taylor series approximations in uncertainty propagation calculations, where higher-order approximations may provide more accurate estimations [15].
3. The approximation of the uncertainty of the prior probability for disease using the Agresti–Coull-adjusted Waldo interval, despite more accurate methods being available [58].
4. The approximations of the sampling uncertainties for both the sample means and standard deviations, which can be improved for smaller samples or pronounced skewness observed in lognormal and gamma distributions [59,60].
5. The use of confidence intervals derived from the *t*-distribution despite the high relative uncertainty [61]. Though not typical in a Bayesian context, this can be employed instead of credible intervals as a practical tool under certain circumstances [5,62].

While addressing these limitations would increase considerably computational complexity, they represent key areas for future enhancement [63,64].

### 5.3. Limitations of the Case Study

The case study’s main limitations include reliance on the OGTT as the reference method for diagnosing diabetes mellitus, despite several factors influencing glucose tolerance [65,66,67,68,69,70,71,72]. Additionally, the lognormal distributions used only approximate the distributions of the FPG measurements from NHANES datasets, highlighting the need for more flexible statistical models.

### 5.4. Challenges in Bayesian Analysis for Disease Diagnosis

While Bayesian analysis may be beneficial in medical diagnostics, it presents certain challenges. For instance, the substantial uncertainty of the posterior probability for disease revealed in our study could lead to clinical indecision. Additionally, there is a notable lack of comprehensive statistical research on the distribution of measurands in both diseased and nondiseased populations, hindering further advancements in Bayesian analysis in this field.

### 5.5. Implications of Incomplete Data

1. Over-reliance on prior probabilities: Limited empirical data may cause an overdependence on prior probabilities, leading to distorted posterior probabilities and potentially flawed clinical decisions [73].
2. Increased uncertainty: Insufficient data amplifies the uncertainty of computed posterior probabilities, which in turn could exacerbate clinical indecision [74].
3. Bias risks: Unrepresentative datasets could introduce systemic bias, increasing the uncertainty in Bayesian computations [5].

### 5.6. Analysis of the Double Sigmoidal Curve in Posterior Probability Estimation and Its Impact on Uncertainty

The posterior probability for disease curve, characterized by a double sigmoidal shape featuring two symmetrical sigmoid functions, presents compelling analytical perspectives in the field of medical diagnostic statistics. This configuration implies that the risk associated with the disease may escalate at both the lower and upper extremes of a given measurand, while a zone of relative safety exists in the intermediate range. Notably, the uncertainty associated with the posterior probability for disease becomes markedly pronounced along the steep segments of the double sigmoidal curve. This heightened uncertainty is attributable to the fact that minor variations in the measurand value can lead to significant alterations in the computed posterior probability.

### 5.7. Software Comparison

Our software easily generates a wide array of parametric plots and comprehensive tables for the analysis of uncertainty of posterior probability. To the best of our knowledge, no exixting software, including all major general or medical or Bayesian statistical and uncertainty quantification software packages (JASP^®^ ver. 0.20.0, Mathematica^®^ ver. 14.0, Matlab^®^ ver. R2023b, MedCalc^®^ ver. 20.2.1, metRology ver. 2023, NCSS^®^ ver. 24.0.0, NIST Uncertainty Machine ver. 2.0.0, OpenBUGS ver. 3.3.0, R ver. 4.3.1, SAS^®^ ver. 9.5, SPSS^®^ ver. 29, Stan ver. 2.33.0, Stata^®^ ver. 19, and UQLab ver. 2.0.0) provides this range of plots and tables without requiring advanced programming.

## 6. Conclusions

The program we have developed represents a novel approach to estimating and analyzing the uncertainty of Bayesian posterior probabilities in disease diagnosis. This tool stands out not only for its innovative capabilities in the field of medical diagnostics but also as a significant educational and research asset. Considering the difficulties and complexities we have outlined, this software offers essential assistance in applying Bayesian methods and dealing with diagnostic uncertainties, thereby enhancing well-informed decision-making.

Looking forward, it seems imperative that future research should focus on improving this method with advanced statistical concepts and empirically validating it with comprehensive test accuracy studies. Such studies are essential to verify the efficacy and reliability of the program in real clinical settings. Additionally, it is necessary to expand its application across a diverse range of diagnostic modalities. Doing so could enable the program to address a broader spectrum of diagnostic challenges, further enhancing its utility and impact on the medical field.

Our research, undertaken alongside our prior work on the uncertainty of diagnostic accuracy measures [19], creates a foundation for understanding uncertainties in diagnostic tests. With this consideration, we would recommend employing our approach in diagnostic accuracy research, aiming at formulating clear guidelines and establishing best practices to effectively integrate such information into clinical practice [48,75,76,77].

Regarding regulatory issues, it is necessary to ensure that the application of the software adheres to the standards set forth by local regulatory authorities. 

The potential of this program seems to be extending beyond its practical implications in medical diagnostics. As an educational resource, it could offer significant opportunities for training in medical statistics, particularly in the understanding of the uncertainty of Bayesian posterior probabilities. Its user-friendly interface, coupled with the depth of its analytical capabilities, makes it an effective learning tool for both aspiring and experienced professionals in the medical community.

In conclusion, the development and refinement of the Bayesian Diagnostic Uncertainty program are pivotal steps towards navigating the complexities of modern medical diagnostics. Its role in enhancing Bayesian diagnostic methods, coupled with its educational benefits, highlights its capability as a supporting tool in the ongoing evolution of medical practice and research.

## Figures and Tables

**Figure 19:**
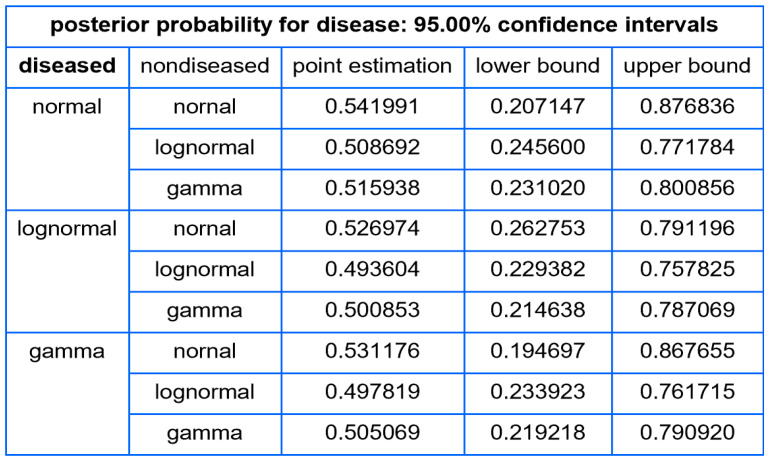
Confidence intervals of the posterior probability for diabetes, with the settings of the program in Table 2.

**Table 1:** Descriptive statistics of the fasting plasma glucose datasets.

	Diabetic Participants	Nondiabetic Participants
*n*	154	822
Mean	120.7	102.6
Median	117.0	102.0
Standard Deviation	19.1	10.9
Skewness	1.448	0.523
Kurtosis	6.354	3.445

**Table 2:** Descriptive statistics of the estimated lognormal distributions of the diabetic and nondiabetic populations.

	Diabetic Participants		Nondiabetic Participants	
Estimated Distribution	$L_{D}$	$l_{D}$	$L_{\bar{D}}$	$l_{\bar{D}}$
Mean Uncertainty	1.586	0	1.028	0
Mean	120.7	120.7	102.6	102.6
Median	119.4	119.4	102.1	102.1
Standard Deviation	17.8	17.7	10.9	10.7
Skewness	0.446	0.444	0.315	0.312
Kurtosis	3.355	3.352	3.177	3.174
*p*-value (Cramér–von Mises test)	0.294	0.295	0.281	0.299

**Table 3:** The settings of the program Bayesian Diagnostic Uncertainty for Figure 5, Figure 6, Figure 7, Figure 8, Figure 9, Figure 10, Figure 11, Figure 12, Figure 13, Figure 14, Figure 15, Figure 16, Figure 17, Figure 18 and Figure 19.

Settings	Figure 5 and Figure 6	Figure 7	Figure 8 and Figure 9	Figure 10	Figure 11 and Figure 12	Figure 13	Figure 14 and Figure 15	Figure 16	Figure 17 and Figure 18	Figure 19
*p*	-	0.95	-	0.95	-	0.95	-	0.95	-	0.95
*x*	31.0–192.0	31.0–192.0	126.0	126.0	126.0	126.0	126.0	126.0	126.0	126.0
$\mu_{D}$	120.7	120.7	120.7	120.7	120.7	120.7	120.7	120.7	120.7	120.7
$\sigma_{D}$	17.7	17.7	17.7	17.7	17.7	17.7	17.7	17.7	17.7	17.7
$n_{D}$	154	154	154	154	154	154	-	-	154	154
$\mu_{\bar{D}}$	102.7	102.7	102.7	102.7	102.7	102.7	102.7	102.7	102.7	102.7
$\sigma_{\bar{D}}$	10.7	10.7	10.7	10.7	10.7	10.7	10.7	10.7	10.7	10.7
$n_{\bar{D}}$	822	822	822	822	822	822	-	-	822	822
*n*	-	-	-	-	-	-	65–5000	65–5000	-	-
*r*	-	-	-	-	-	-	0.158	0.158	-	-
$b_{0}$	0.866	0.866	0.0–0.161	0.0–0.161	0.866	0.866	0.866	0.866	0.866	0.866
$b_{1}$	0.0109	0.0109	0.0109	0.0109	0.0–0.1	0.0–0.1	0.0109	0.0109	0.0109	0.0109
$n_{U}$	-	1350	-	1350	-	1350	-	1350	-	1350
$l_{D}$	lognormal	lognormal	lognormal	lognormal	lognormal	lognormal	lognormal	lognormal	normal lognormal gamma	normal lognormal gamma
$l_{\bar{D}}$	lognormal	lognormal	lognormal	lognormal	lognormal	lognormal	lognormal	lognormal	normal lognormal gamma	normal lognormal gamma

## Data Availability

The data presented in this study are available at https://wwwn.cdc.gov/nchs/nhanes/default.aspx (accessed on 20 December 2023).

## Supplementary Materials

The following supporting information can be downloaded at: Supplementary File S1: BayesianDiagnosticUncertainty.nb: The program as a Wolfram Mathematica Notebook. Available at https://www.hcsl.com/Tools/BayesianDiagnosticUncertainty/BayesianDiagnosticUncertainty.nb (accessed on 4 February 2024); Supplementary File S2: BayesianUncertaintyCalculations.nb: The calculations for the estimation Bayesian posterior probability for disease and its standard uncertainty in a Wolfram Mathematica Notebook. Available at https://www.hcsl.com/Supplements/SBDU.zip (accessed on 4 February 2024); Supplementary File S3: BayesianDiagnosticUncertaintyInterface.pdf: A brief description of the interface of the program. Available at: https://www.hcsl.com/Documents/BayesianDiagnosticUncertaintyInterface.pdf (accessed on 4 February 2024).

## Appendix A

### Appendix A.1. Formalisms and Notation

Acronyms

PDF: probability density function

FPG: fasting plasma glucose

OGTT: oral glucose tolerance test

NHANES: National Health and Nutrition Examination Survey

Notation

Parameters

$n_{D}$ : size of diseased population

$\mu_{D}$ : mean of diseased population

$\sigma_{D}$ : standard deviation of diseased population

$n_{\bar{D}}$ : size of nondiseased population

$\mu_{\bar{D}}$ : mean of nondiseased population

$\sigma_{\bar{D}}$ : standard deviation of nondiseased population

r : prior probability for disease (prevalence rate)

$u_{s}\left(x\right)$ : standard sampling uncertainty of x

$u_{m}\left(x\right)$ : standard measurement uncertainty of x

$u_{c}\left(x\right)$ : standard combined uncertainty of x

$n_{U}$ : number of quality control measurements

$b_{0}$ : constant contribution to measurement uncertainty

$b_{1}$ : measurement uncertainty proportionality constant

$\hat{u}$ : mean standard measurement uncertainty

p : confidence level

$v_{eff\;}$ : effective degrees of freedom

Functions

$P\left(A\right)$ : probability of the event *A*

$P\left(A|B\right)$ : conditional probability of the event *A* given the event *B*

$L\left(\theta |z\right)$ : likelihood function

$F^{-1}\left(.\right):$ the inverse function *F* $\;\left(.\right)$

Bayes’ Theorem

For the purposes of our study, Bayes’ theorem is formulated as:

$$P\left(D|T\right)=\frac{P\left(T|D\right)P\left(D\right)}{P\left(T\right)}=\frac{P\left(T|D\right)P\left(D\right)}{P\left(T|D\right)P\left(D\right)+P\left(T|\bar{D}\right)\left(1-P\left(D\right)\right)}$$

where

$P\left(D|T\right)$ denotes the posterior probability of having a disease D given a test result T .

$P\left(T|D\right)$ denotes the likelihood of obtaining the result T given the presence of the disease D .

$P\left(T|\bar{D}\right)$ denotes the likelihood of obtaining the result T given the absence of the disease D .

$P\left(D\right)$ is the prior probability or prevalence r of the disease D .

$P\left(T\right)$ is the overall probability of the result T .

According to Bayes’ theorem, the posterior probability for a disease D given a test result T=x and a parameter vector ***θ*** is calculated as:

$$P\left(D|T\right)=\frac{L_{D}\left(\theta |x\right)r}{L_{D}\left(x|\theta \right)r+L_{\bar{D}}\left(x|\theta \right)\left(1-r\right)}=\frac{f_{D}\left(x|\theta \right)r}{f_{D}\left(x|\theta \right)r+f_{\bar{D}}\left(x|\theta \right)\left(1-r\right)}$$

where r denotes the prior probability for disease, $L_{D}\left(\theta |x\right)$ and $f_{D}\left(x|\theta \right)$ denote the likelihood function and the PDF of the test measurand in the presence of the disease, respectively, while $L_{\bar{D}}\left(x|\theta \right)$ and $f_{\bar{D}}\left(x|\theta \right)$ are the respective functions in the absence of the disease.

#### Appendix A.1.1. Parametric Distributions

It is assumed that the test measurands of the diseased or nondiseased populations follow the normal, lognormal or gamma distribution. The domains of random variables for the respective distributions are defined as follows:

1. The domain of a random variable *X* following a normal distribution is the set of all real numbers, denoting −∞<X<∞ .
2. The domain of a random variable *X* following a lognormal distribution is the set of all positive real numbers, denoting 0<X<∞ .
3. The domain of a random variable *X* following a gamma distribution is the set of all positive real numbers, denoting 0<X<∞ .

#### Appendix A.1.2. Calculations of the Posterior Probability for Disease and Its Uncertainty

These calculations are detailed in Supplementary File S2 (Refer to Supplementary Files).

### Appendix A.2. Software Availability and Requirements

Program name: Bayesian Diagnostic Uncertainty

Version: 1.0.0

Project home page: https://www.hcsl.com/Tools/BayesianDiagnosticUncertainty/ (accessed on 4 January 2024)

Available at: https://www.hcsl.com/Tools/BayesianDiagnosticUncertainty/BayesianDiagnosticUncertainty.nb (accessed on 4 January 2024)

Operating systems: Microsoft Windows 10+, Linux 3.15+, Apple macOS 11+

Programming language: Wolfram Language

Other software requirements:

For running the program and reading the BayesianDiagnosticUncertaintyCalculations.nb file Wolfram Player^®^ ver. 12.0+ is required, freely available at: https://www.wolfram.com/player/ (accessed 18 December 2023) or Wolfram Mathematica^®^ ver. 12.0+

System requirements: Intel^®^ i9™ or equivalent CPU and 32 GB of RAM

License: Attribution—Noncommercial—ShareAlike 4.0 International Creative Commons License

### Appendix A.3. A Note about the Program

About the Program Controls

The program features an intuitive tabbed user interface, designed to streamline user interaction and facilitate effortless navigation across its multiple modules and submodules.

The numerical settings are defined by the user with menus or sliders. Sliders can be finely manipulated by holding down the *alt* key or *opt* key while dragging the mouse. They be even more finely manipulated by also holding the *shift* and/or *ctrl* keys.

Dragging with the mouse while pressing the *ctrl*, *alt*, or *opt* keys zooms plots in or out.

Range of input parameters

$x:\;maximum\left(\mu_{\bar{D}}-5\sigma_{\bar{D}},0\right)-\mu_{D}+5\sigma_{\bar{D}}$

$n_{D}$ : 2–10,000

$\mu_{D}$ : 0.1–10,000

$\sigma_{D}$ : 0.01–1000

$n_{\bar{D}}$ : 2–10,000

$\mu_{\bar{D}}$ : 0.1–10,000

$\sigma_{\bar{D}}$ : 0.01–1000

r : 0.010–0.500

$n_{U}$ : 20–10,000

$b_{0}$ : 0– $\sigma_{\bar{D}}$

$b_{1}:$ 0–0.1

p : 0.900–0.999
