# Correction: Chatzimichail, T.; Hatjimihail, A.T. A Software Tool for Estimating Uncertainty of Bayesian Posterior Probability for Disease. *Diagnostics* 2024, *14*, 402

**DOI:** 10.3390/diagnostics14131354

**Published:** 2024-06-26

**Authors:** Theodora Chatzimichail, Aristides T. Hatjimihail

**Affiliations:** Hellenic Complex Systems Laboratory, Kostis Palamas 21, 66131 Drama, Greece; tc@hcsl.com

There was an error in the original publication [1]. The value of the term $b_{1}$ of the function relating the standard measurement uncertainty, $u_{m}\left(x\right)$, to the measurement value, *x*, was stated as being equal to 0.109 instead of 0.0109.

A correction has been made to Section 3, ‘Illustrative Case Study’, paragraph number 8:

The NHANES quality control data of the FPG measurements was retrieved for the same period (2005–2016). In total, 1350 QC samples had been analyzed. The weighted nonlinear least squares analysis [36] yielded the following function relating the standard measurement uncertainty $u_{m}\left(x\right)$ to the measurement value, *x*:

$$u_{m}\left(x\right)=\sqrt{b_{0}^{2}+b_{1}^{2}x^{2}}=\sqrt{0.7501+0.00012x^{2}}$$

where $b_{0}=0.866$ and $b_{1}=0.0109$.

The authors state that the scientific conclusions are unaffected. This correction has been approved by the Academic Editor. The original publication has also been updated.
